# A Modified Approach for the Ultrasound-Guided Quadratus Lumborum Block in Dogs: A Cadaveric Study

**DOI:** 10.3390/ani11102945

**Published:** 2021-10-12

**Authors:** Jaime Viscasillas, Jose Terrado, Reyes Marti-Scharfhausen, Diego Castiñeiras, Vicente Esteve, Niamh Clancy, Jose Ignacio Redondo

**Affiliations:** 1 Departamento Medicina y Cirugía Animal, Facultad de Veterinaria, Universidad Cardenal Herrera-CEU, CEU Universities, 46115 Valencia, Spain; jterrado@uchceu.es (J.T.); maria.marti29@uchceu.es (R.M.-S.); vicente.esteve@uchceu.es (V.E.); nacho@uchceu.es (J.I.R.); 2 Willows Veterinary Centre & Referral Service, Highlands Rd, Shirley, Solihull B90 4NH, UK; dfcastineiras@hotmail.com; 3 The Royal Veterinary College, Hawkshead Lane, North Mymms, Hatfield AL9 7TA, UK; nclancy@rvc.ac.uk

**Keywords:** canine, quadratus lumborum, regional anaesthesia, ultrasound

## Abstract

**Simple Summary:**

This study describes a modified approach for the ultrasound-guided quadratus lumborum block in dogs. Previous studies carried out in canine cadavers describe the needle insertion following a ventro-lateral to dorso-medial approach. Our modified technique follows a dorso-lateral to ventro-medial direction. We aimed to have the same success with this approach as previous studies in dogs but to minimise the potential complications. After performing the modified technique bilaterally in nine canine cadavers and administering contrast, we assessed the contrast distribution with computed tomography (CT) and dissection. Potential complications were also assessed. Our dissection results showed similar distribution to previous studies, although CT results showed a more caudal contrast spreading. No contrast was found in the abdomen or epidural space. This study shows that our modified approach is safe and has at least the same distribution as the previous studies published in dogs.

**Abstract:**

Ultrasound-guided quadratus lumborum block (QLB) is a locoregional technique described in canine cadavers. The aim of this study was to assess a modified approach to QLB to minimise potential complications such as abdominal organ puncture. Nine canine cadavers were included and were positioned in lateral recumbency. An ultrasound-guided QLB was performed on each side. The probe was placed in the transverse position over the lumbar muscles just caudal to the last rib, and a needle was advanced in-plane from a dorso-lateral to a ventro-medial. A volume of 0.2 mL kg^−1^ of a mixture of iomeprol and methylene blue was injected. Computed tomography (CT) and dissection were performed to evaluate the spreading. Success was defined as staining of the nerve with a length of more than 0.6 cm. Potential complications such as intra-abdominal, epidural, or intravascular spreading of the mixture were also assessed. The CT images showed a T13 to L7 vertebra distribution, with a median of 5 (3–6). Dissection showed staining of the nerves from T13 to L4, with a median of 3 (2–5). No complications were found. This modified approach to QLB is safe and shows similar results to the previous studies in canine carcass.

## 1. Introduction

The ultrasound (US)-guided quadratus lumborum block (QLB) is a regional anaesthesia technique first described in humans by Blanco et al. as a posterior (dorsal) transversus abdominis plane (TAP) block [1]. Due to the spread of the local anaesthetic (LA) into quadratus lumborum (QL) plane and ventral thoracic paravertebral space however, QL block (QLB) is used to provide superior abdominal pain control to TAP block as wall analgesia and visceral analgesia are both provided [2,3]. In human anaesthesia, the QLB has been described for perioperative pain management in patients undergoing abdominal [4,5,6] and pelvic limb surgery [7]. Therefore, this technique is a potential alternative to neuraxial anaesthesia in both scenarios. Since Blanco et al. [1] first described the technique to perform the QLB, in human anaesthesia, different approaches to the QL, mainly in terms of the location of the tip of the needle and the consequent pattern of LA distribution, have been developed [8]

The main differences among them are the location of the tip of the needle and the distribution of local anaesthetic. To the authors’ knowledge, none of these techniques have been demonstrated to provide better analgesia or block a more extensive area.

In dogs, the quadratus lumborum (QL) muscle lies directly ventral to the bodies of the last three thoracic and all the lumbar vertebrae. It also lies ventral to the proximal portions of the last two ribs and the transverse processes of the lumbar vertebrae. The area caudal to the first lumbar vertebra is covered ventrally by the psoas minor muscle and caudal to the fourth lumbar vertebra by the major muscle. It ends on the medial surface of the wing of the ilium between the articular surface and the cranial ventral iliac spine. The lateral portion of the muscle overhangs the transverse processes of the lumbar vertebrae so that it also comes to lie on the ventral surface of the tendon of origin of the transversus abdominis muscle [9]. The last thoracic and the first three lumbar nerve roots exit from the vertebral foramen and lie in the subserous endothoracic fascia at its origin. These nerves run between the QL and psoas muscles then finally pass through the aponeurosis of origin of the transversus abdominis muscle. Therefore, the injection of local anaesthetic between the QL and psoas muscles would theoretically block these nerves.

In the veterinary literature, two cadaveric studies have been published in dogs describing different approaches to carry out the QLB. In both, the administration of contrast between the QL and psoas muscles [10] and lateral to the QL [11] showed consistent staining of the nerves and sympathetic trunk from T13 to L3. In both studies, the needle was advanced from the abdominal wall to reach the injection point. Therefore, the needle passes through the obliquus externus and internus abdominis muscles; the aponeurosis of insertion of the transversus abdominis muscle; and, at this point, either the tip of the needle is placed lateral to the QL muscle, or it passes through the QL muscle to reach the fascia between QL and psoas muscles. One recognised potential complication of QLB in human anaesthesia is the puncture of abdominal organs [8,12] due to the proximity kidneys, spleen, or even liver to the QL and psoas muscles. To author’s knowledge, this complication has not been reported in dogs, however, since the abdominal wall is very thin in some dogs, and needle handling might be complex in some animals.

The aim of this study was to develop a dorsal approach to the QLB and describe the sonoanatomy to perform the QLB, where the needle is introduced through the epaxial muscles and advanced until the tip of the needle is placed between QL and psoas muscle.

In addition, the spreading with the pattern of a mixture of dye and radiological contrast distribution obtained was evaluated by anatomical dissections and advance imaging technique. 

Our hypotheses are this modified approach will have an equivalent pattern of distribution to previous canine studies [10,11] and that undesirable locations of the contrast, such as epidural space or abdomen, will not be found. 

## 2. Materials and Methods

This study was performed at the Universidad CEU Cardenal Herrera in Valencia (Spain), following the European (2010/63/UE) and Spanish (RD 53/2013) laws regarding experimentation on animals. Nine thawed canine carcasses, euthanised for reasons not related to this study, were included. This number was based on previous cadaveric studies published in dogs [10]. The carcasses were kept in the freezer between two weeks and one month before the experiment was performed. The carcasses were left two days at room temperature before the experiment was performed. 

All carcasses were placed in lateral recumbency to perform the injections, and the QLB technique was carried out on the uppermost side. The abdomen of the carcasses was clipped, and ultrasound gel was applied. The technique was carried out twice in each carcass with one injection per side. The order of these injections was randomised using the free software www.randomize.org with both the right- and left-sided injections being administered the same way. Only one anaesthetist performed all the injections using a 22-gauge, 88 mm, or 22-gauge, 35 mm Quincke needle (Spinocan; BBraun, Melsungen, Germany), depending on the body weight of the carcass. The longer needles were used for dogs larger than 10 kg. A linear 13–6 MHz ultrasound probe (Sonosite HFL38/13-6 for MicroMaxx Ultrasound Probe; Sonosite, Bothell, WA, USA) attached to an ultrasound machine (Edge II; Sonosite, WA, USA) was used on all the carcasses. The needle was connected to an extension set (Discofix C; BBraun) and a syringe (Omnifix; BBraun). A total volume of 0.2 mL kg^−1^ of a 50:50 mixture of iomeprol (Iomeron; Bracco Imaging, Milan, Italy) and methylene blue (methylthioninium chloride injection 1% *w*/*v*; Martindale Pharmaceuticals, Romford, UK) was administered in each injection. 

**Figure 1:**
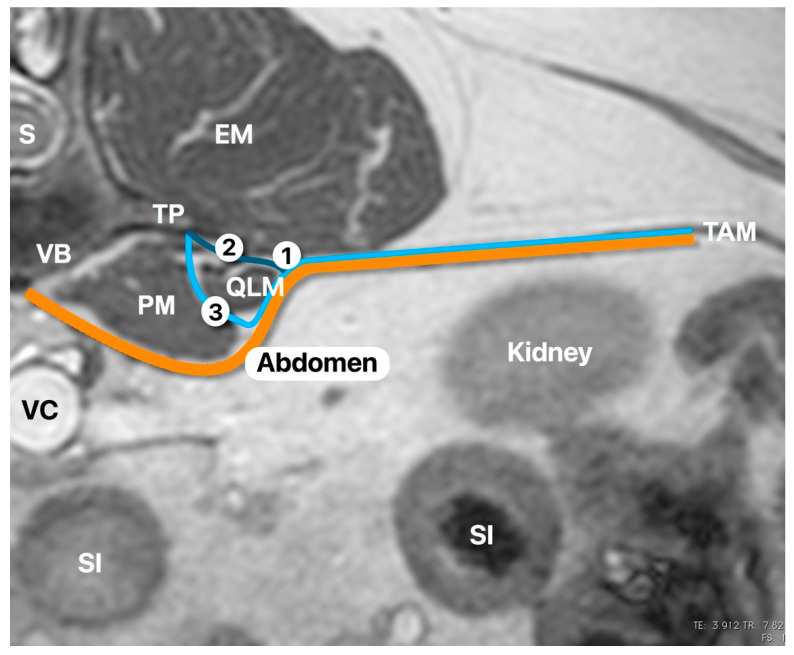
Transverse view of a canine abdominal MRI at the level of the first lumbar vertebrae (L1) to illustrate the anatomy. The picture shows the relevant anatomy related to the quadratus lumborum block (QLB) and the different approaches described in the human literature to perform the QLB. PM, psoas muscle; QLM, quadratus lumborum muscle; TP, transverse process; VB, vertebral body; S, spine; EM, epaxial muscles; TAM, transversus abdominis muscle; VC, vena cava; SI, small intestine. The orange line is the fascia transversalis. The light blue line is the anterior thoracolumbar fascia. The dark blue line is the middle thoracolumbar fascia (based on three-layered model of the thoracolumbar fascia [7]). (1) Point of injection of the QLB1 technique; (2) point of injection of the QLB2 technique; (3) point of injection of the QL3 technique. Picture was taken and modified by the author without any copyright dispute.

**Figure 2:**
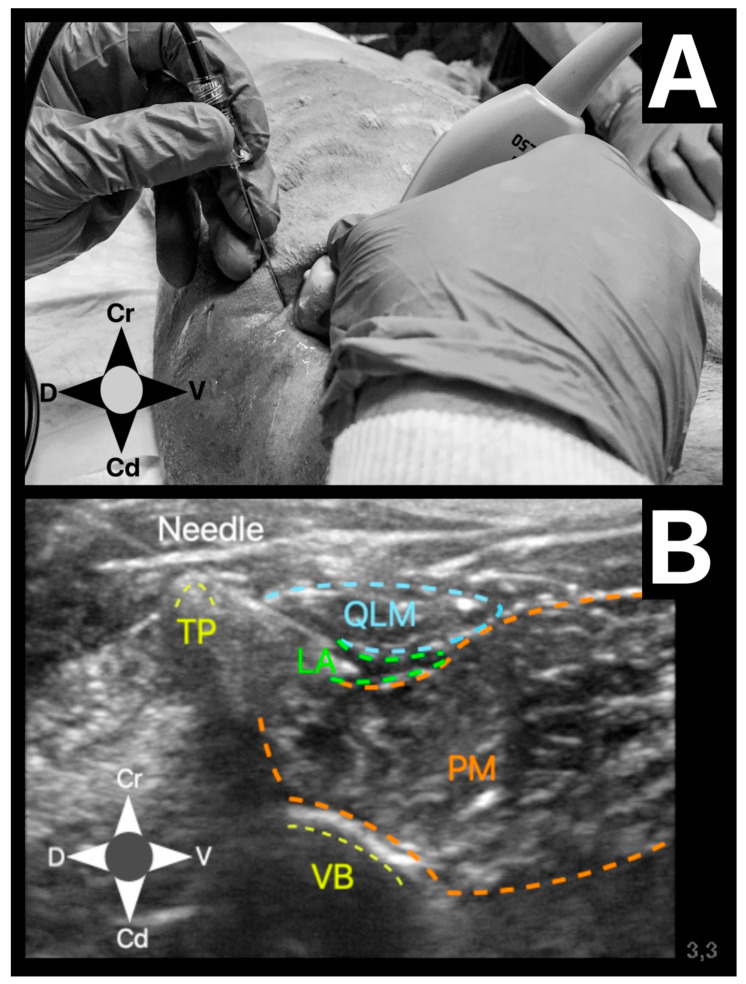
(**A**) Showing the positioning of the ultrasound probe and needle using the in-plane technique. The ultrasound probe was positioned in the lateral aspect of the lumbar muscles, caudal to the last rib, to perform the quadratus lumborum block. Cr, cranial; Cd, caudal; D, dorsal; V, ventral. (**B**) Ultrasound image showing the landmarks of the QLB. TP (yellow), transverse process; VB (yellow), vertebral body; QLM (blue), quadratus lumborum muscle; PM (orange), psoas muscle; LA (green), local anaesthetic between quadratus lumborum and psoas muscle.

**Figure 3:**
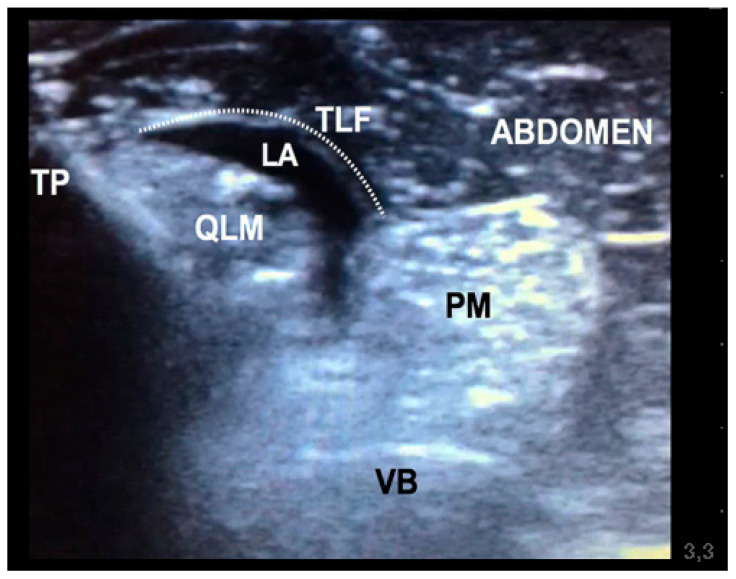
Ultrasound image obtained after injection in a canine carcass during quadratus lumborum block (QLB). The picture shows hydrodissection of the quadratus lumburom muscle from the psoas muscle and the thoracolumbar fascia, illustrating that the QLB block was successful. TP, transverse process of L2 vertebrae; VB, vertebral body of L2 vertebrae; QLM, quadratus lumborum muscle; PM, psoas muscle; LA, mixture of contrasts; TLF, thoracolumbar fascia.

The US-guided QLB technique was performed in the following manner. The US probe was placed in a transverse plane over the lumbar muscles just caudal to the last rib using a paramedian sagittal oblique (subcostal) approach described by Elsharkawy [13] in humans. Once in position, the probe was rotated slightly cranial to visualise the following anatomical structures: quadratus lumborum muscle, psoas muscle, lateral aspect of the body of the first lumbar vertebrae, and the transverse process of the first lumbar vertebrae (Figure 1). The needle was inserted in-plane and advanced at a 45º angle from dorsolateral to ventromedial until its tip was placed between the quadratus lumborum and psoas muscles (Figure 2). At this location, the mixture was injected into the fascial plane. A volume of less than 1 mL of saline was first injected to confirm the correct position of the tip of the needle. The injection was considered as correct when hydrodissection of the quadratus and psoas muscles together with the ventral movement of the thoracolumbar fascia was noted (Figure 3). If this pattern was not visualised, the needle was redirected until this hydrodissection was observed.

The CT images were taken five minutes after the anaesthetist had performed both injections in each animal. The scanner employed was a Brivo CT 385 (GE Hangwei Medical Systems Co., Ltd.; Beijing, China) set at 120 Kv, 199 mA, and 1.25 mm slide thickness parameters. A 3D reconstruction of the CT images was performed using Horos DICOM Medical Image Viewer (Horos Project, 2015), and a veterinary radiologist assessed the images. The parameters evaluated on CT images were the extension and pattern of contrast distribution based on the number of vertebrae.

After obtaining the CT images, we moved the carcasses to the post-mortem room, where another investigator, different from the previous one, performed a dissection, unaware of the CT images. An incision was made through the linea alba into the abdominal cavity; the digestive tract, spleen, and pancreas were eliminated en bloc by sectioning at the cardiac and rectal levels. Subsequently, the parietal peritoneum was incised, and the ventral roots of the lumbar nerves were dissected. This was performed by separating the two muscle segments of the quadratus lumborum (to expose L1) or by dissecting the bellies of the psoas major and quadratus lumborum to dissect the ventral branches of the rest of the lumbar nerves. The parameter evaluated on dissection was the staining of T13, L1, L2, L3, L4, L5, L6, and L7 nerves. Success was defined as staining of the nerve with a length of more than 0.6 cm [14]. Potential complications such as distribution of mixture into the epidural space, abdominal cavity, or intravascular space were assessed by CT images and dissection. 

The data were analysed using R 3.4.0, a free software environment for statistical computing and graphics (R Core Team, 2017). They were reported as numbers of cases (n/n) and percentage (%). Median (range) was also calculated. A Fisher’s exact test was used to compare the number of observations for the stained nerves and the injection spread between sides (right and left). A *p*-value < 0.05 was considered statistically significant. 

## 3. Results

Canine carcasses used in our study belonged to different breeds (one Yorkshire terrier, one Siberian husky, one German shepherd dog, and six crossbreeds) and body weights, with a median of 15 kg (2.7–45 kg). Body condition score was 4 (3–7) according to the AAHA nutritional assessment guidelines for dogs and cats [15]. All the animals were adult dogs.

**Figure 4:**
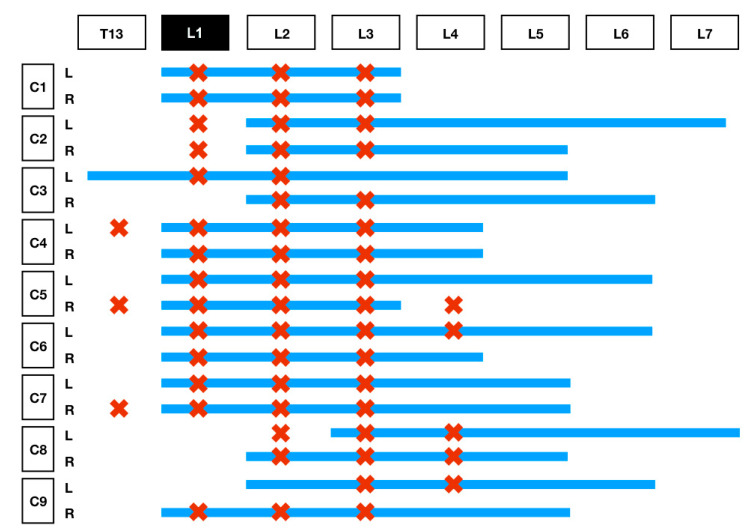
This table represents the difference in the extent of diffusion of the 50:50 iohexol/methylene blue mixture observed during CT versus dissection. The *Y*-axis of the graph represents the spread of the mixture from T13 to L7 using the red Xs to show the spread noted on the ventral branches of the nerve roots on dissection, while the blue line represents the distribution around the vertebral bodies seen on CT. The *X*-axis represents the 9 carcasses used in this study, comparing the left- and right-sided blocks.

**Figure 5:**
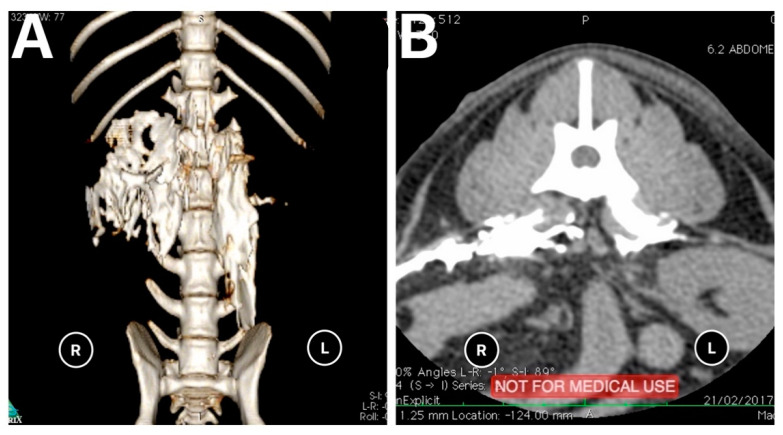
(**A**). 3D CT image of a carcasses showing a combined linear-lateral pattern of distribution of the mixture on the right and linear pattern on the left after performing the quadratus lumborum block. (**B**). Transverse view of CT image showing a lateral pattern on the right and linear pattern on the left.

The results of the distribution of the mixture in the CT images and dissection are summarised in Table 1 and Figure 4. On the basis of the CT images, we found that 4/18 (22.3%) injections showed a linear and lateralised pattern (Figure 5). Therefore, the mixture was visualised from the thoracolumbar fascia and moved towards the transversus abdominis plane. Conversely, 13/18 (72.3%) injections followed a linear pattern, and the mixture was always found between the quadratus lumborum and psoas muscle. No statistical differences were found between the distribution pattern or whatever side was used to spread the mixture, neither in CT images nor in dissection (*p* = 1). 

Only 1/18 (0.05%) injection was found inside the psoas muscle, for which both CT and dissection were confirmed. This injection was correlated with the low spreading of the mixture. 

CT images showed 0/18 injections in unwanted locations such as inside the abdominal cavity, epidural space, or intravascularly.

## 4. Discussion

The dorsal US-guided approach to QLB described in this study appears to be a feasible technique in dogs. All the injections but one were located between the QL and psoas muscle, demonstrating that this technique can be easily reproduced in dogs. However, it is essential to mention that visualisation of the fascia between both muscles was complex in some carcasses, and the probe was angled in several positions until the operator achieved an adequate visualisation of both anatomical structures. Interestingly, contrast was found inside the psoas muscle in just one injection. This result might be related to the use of a low volume of saline to confirm the correct location of the tip of the needle before contrast administration. However, we cannot rule out that using thawed instead of fresh carcasses or living specimens might have also had an impact on our results.

The pattern of distribution (linear +/− lateralised) found in our study could be related to the injection point. This is compatible with the different approaches described in human anaesthesia [16,17]. The QLB1 (or lateral approach) involves administering the local anaesthetic inside the triangle formed by the lateral aspect of the QL muscle, internal aspect of the transversus abdominins muscle, and the anterior thoracolumbar fascia. This approach will produce a lateral distribution of the local anaesthetic. With the QLB2 (or posterior approach), the needle is aimed at the dorsal aspect of the QL muscle, and local anaesthetic is administered inside the middle thoracolumbar fascia. This approach will produce a linear distribution dorsal to the QL muscle. The QLB3 (or transmuscular approach) inserts a needle through the quadratus lumborum and injects local anaesthetic between QL and the psoas muscles. This approach will separate both muscles and will produce a linear and slightly lateral distribution since the anterior thoracolumbar fascia is in direct contact with the ventral border of both muscles [16,18]. Finally, the QL intramuscular approach involves advancing the needle tip until it penetrates the fascia and it is then inserted into the QL muscle [2]. In our study, the QLB3 was utilised in all the cases, and CT images showed a linear and slightly lateral distribution pattern compatible with the QLB3 approach. It is still unclear as to which approach would be more advantageous regarding the authors’ knowledge based on the human literature. There might be some clinical differences since the QLB3 approach appears to spread more caudally but less cranially than the other approaches [2]. Garbin et al. [10,11] found more cranial spreading using a QLB1 versus QLB3 approach in their studies in dogs. However, clinical studies are needed in veterinary medicine to confirm these results.

We found some differences between the results obtained with CT images in comparison with those from the dissection because they reveal different aspects of the technique. Dissection showed which nerves were actually stained. Conversely, CT images indicated the extension and distribution of the injection along with the vertebral bodies and therefore provided us with a better understanding of how the local anaesthetic might diffuse once injected between both muscles. Therefore, the dissection and CT images obtained in the current study provided valuable information about how the local anaesthetic might distribute in living animals. However, it is crucial to consider the tissue differences in a thawed carcass to a living specimen.

Our study’s results regarding the spreading of dye during the dissection are similar to those reported in another canine study with the same injection point [9]. Therefore, our modified approach to the QLB does not seem to affect the potential efficacy of the technique. 

The CT images showed a caudal distribution of the contrast mixture starting from the level of L2 in most cases and reaching L7 in some injections. However, no nerves were stained during the dissection caudally to L4. Garbin et al. [9] studied the contrast distribution with MRI in two carcasses after carrying out an US-guided QLB, but they did not report the same caudal spreading. In their study, contrast ended up on L4/L5 while 12/18 (66%) reached at least L5 in our study. The reason for this difference could simply be the higher number of carcasses we used in our study. On the contrary, although CT images showed contrast starting from the level of L2, the dissection showed staining of the nerves starting at T13 in 3/18 (16.7%) of the injections. This difference could be explained anatomically by the caudolateral direction the ventral roots of the thoracic and lumbar nerves take when exiting the intervertebral foramen. 

Our CT results showed a precise caudal distribution of the mixture in most cases, with only one injection reaching the cranial part of the T13 vertebra. This finding is in agreement with the results found by Carline et al. [13]; however, it is in contrast to the cranial distribution obtained in other human cadaveric studies [19] or canine cadaveric study [11], in which the contrast reached a more proximal area (T11). It is not clear why this cranial distribution does not occur on the basis of canine anatomy. The QL muscle reaches the 10th or 11th thoracic vertebrae in dogs; therefore, it should be possible for the mixture to extend to that location. One potential explanation could be that the needle was directed caudally in all cases, which might explain why the distribution was only in a caudal direction since the needle trajectory may influence the spread and clinical effect of the block [20]. Finally, another potential reason could be the volume administered in this study. The volume used in our study was based on the one administered in the study by Carline et al. [16]. Canine cadaveric studies have used between 0.15 and 0.3 mL kg^−1^. Other authors have used up to 0.5 mL kg^−1^ of ropivacaine 2% on clinical cases in humans [20]. More studies are needed to evaluate the spreading of contrast with the needle facing cranially and/or increasing the injection volume. 

The CT images showed radiographic contrast distribution at the L4, L5, and L6 vertebrae levels in 83%, 61%, and 33% of the injections, respectively. However, no methylene blue was found upon dissection of those nerves. Our CT distribution findings agree with some studies in human anaesthesia [16]. In a study conducted by Winker [21], a patient was found to have slight femoral nerve deficits after a US-guided QLB was performed for an abdominal procedure. Therefore, it is reasonable to think this technique might cause caudal distribution in some cases and block the lumbar plexus. Some published work has even shown the use of QLB as a new approach for the lumbar plexus block [6,7,21,22,23]. On the basis of the results of this study, we cannot provide a clear explanation about how the local anaesthetic reaches these nerves once they are inside the psoas muscle. One potential explanation could be the spreading of local anaesthetic into the epidural space, although our results do not support this. Further studies with living specimens could help us understand whether local anaesthetic can diffuse into the psoas muscle and block the lumbar plexus.

The main advantage of the QLB versus TAP block is the addition of visceral analgesia; however, the exact mechanism is still unknown. Some authors hypothesise that the thoracolumbar fascia has a high-density network of sympathetic fibres and mechanoreceptors that might be responsible for the visceral analgesia [8,24,25]. On the other hand, other authors suggest that local anaesthetic might spread to the celiac ganglion or sympathetic chain, as previously described with a paravertebral block via the splanchnic nerves [26,27]. Unfortunately, our study cannot answer that question, and further studies are needed to correlate the distribution of the local anaesthetic and extension of visceral analgesia.

Finally, potential complications were recorded neither during the dissection nor CT images. Abdominal organ puncture (i.e., kidney) has been reported as a potential complication [8]. The authors believe that this scenario is less likely with this approach because the needle is visualised in-plane all the time, and it must pass through all the epaxial muscles and psoas or QL muscles to reach the abdomen. Therefore, the operator has more time to notice the correct needle angle and can advance the needle slowly until the tip is between QL and psoas muscles. Epidural injection/contamination has also been mentioned in the literature [10,21]. Again, our modified approach advanced the needle from dorso-lateral to ventro-medial. Therefore, the needle is moving away from the intervertebral foramen, and the administration of the local anaesthetic is in the opposite direction. It is then difficult to either advance the needle into the vertebral canal or spread the local anaesthetic to the vertebral foramen.

Our study has some limitations. Only one volume of mixture was administered so we cannot assess whether a higher volume would increase the cranial and caudal spreading. In addition, there is an absence of a comparison group where the approaches described by Garbyn et al. [10,11] were performed. Therefore, although we did not found contrast in undesirable locations, it is impossible to assess that the modified approach is safer. Finally, the viscosity of the radiographic contrast is higher than local anaesthetics; therefore, more spreading might be expected using just local anaesthetics. Marhofer et al. [15] reported a wider distribution area for sensory blocks than expected according to MRI images obtained after injecting a mixture of radiographic contrast and local anaesthetic. Therefore, to evaluate the clinical effect of this block, we must assess the technique in living specimens using a local anaesthetic solution.

## 5. Conclusions

The described modified US-guided QLB is a technique that can be performed in dogs. This cadaveric study showed that the distribution of contrast is similar to the previous described approaches in dogs, and therefore it is an alternative. However, further studies are needed to determine the extent of the block in living animals.

## Figures and Tables

**Table 1:** The spreading of a 50:50 iohexol/methylene blue mixture after application of the quadratus lumborum block (QLB) in nine canine carcasses bilaterally (18 injections). These results are based upon CT images and dissection and are shown as number (n/n) and percentage (%). The CT images assessed spreading around the vertebral bodies, while dissection assessed the number of stained ventral branches of the spinal nerves. M (median) represents the spreading of the mixture and R (range) shows the minimum and maximum spread observed. C (complications) shows the complications found with both methods of assessment.

	T13	L1	L2	L3	L4	L5	L6	L7	M (R)	C
CT	1/18 (5%)	12/18 (66%)	17/18 (94%)	18/18 (100%)	15/18 (83%)	11/18 (61%)	6/18 (33%)	2/18 (11%)	5 (3–6)	none
Dissection	3/18 (17%)	14/18 (72%)	17/18 (95%)	17/18 (95%)	5/18 (28%)	0	0	0	3 (2–5)	none

## Data Availability

Data supporting the reported results can be sent to anyone interested by contacting the corresponding author.
